# Polysaccharides of St. John's Wort Herb Stimulate NHDF Proliferation and NEHK Differentiation via Influence on Extracellular Structures and Signal Pathways

**DOI:** 10.1155/2012/304317

**Published:** 2012-07-17

**Authors:** S. Abakuks, A. M. Deters

**Affiliations:** Institute for Pharmaceutical Biology and Phytochemistry, Westphalian Wilhelms University of Münster, Hittorfstra Be 56, 48149 Münster, Germany

## Abstract

St. John's Wort herb extracts often contain undesirable or volitional polysaccharides. As polysaccharides exhibit structure-dependent biological functions in the present study water-soluble polysaccharides were extracted from herb material, fractionated by anion exchange chromatography into four main polysaccharide fractions (denominated as Hp1, Hp2, Hp3 and Hp4) and characterized by HPAEC-PAD, CE, IR and GC-MS. Biological activity on human skin keratinocytes and fibroblasts was assessed by investigation of their effect on proliferation, metabolism, cytotoxicity, apoptosis and differentiation. The underlying mechanisms were investigated in gene expression studies. Polysaccharide fraction Hp1 was mainly composed of **β**-D-glucose. Hp2, Hp3 and Hp4 contained pectic structures and arabinogalactan proteins varying in composition and quantity. Polysaccharides of Hp1 induced the keratinocyte differentiation by inhibiting the gene expression of the epidermal growth factor and insulin receptor. While the collagen secretion of fibroblasts was stimulated by each polysaccharide fraction only Hp1 stimulated the synthesis. The fibroblast proliferation was reduced by Hp1 and increased by Hp4. This effect was related to the influence on genes that referred to oxidative stress, metabolism, transcription processes and extracellular proteins. In conclusion polysaccharides have been shown as biologically active ingredients of aqueous St. John's Wort extracts with a relation between their structural characteristics and function.

## 1. Introduction

Extracts of St. John's Wort (*Hypericum perforatum* L., Hypericaceae) are well investigated and widely used in regard to their effectiveness against moderate depressions. The responsible naphthodianthrones, phloroglucinol derivates, and flavonoids were extensively investigated [1]. Concerning biological activity on the skin the investigations focused on the phototoxicity of lipophilic and aqueous-ethanolic extracts for many years. Beside the ongoing discussion concerning the phototoxicity of St. John's Wort extracts [2, 3], it has been reported that hyperforin induced keratinocyte differentiation *in vitro* and the skin hydration *in vivo* [4, 5]. Nevertheless more information is needed concerning the less investigated side products of extraction. Especially the carbohydrates that conflict with formulation, development, and production of solid dosage forms [6] were mostly seen as nonactive byproducts. There are some reasons for investigation of St. John's Wort polysaccharides. First of all no information is available concerning composition and structure of polysaccharides in *Hypericum* species and cognate plants. Again aqueous-ethanolic extracts of St. John's Wort possess high viscosity indicating coextraction of plant polysaccharides. According to their structural characteristics, herbal polysaccharides are able to influence the immunologic response [7], bacterial adhesion [8], and tumor inhibition [9]. Furthermore they promote the tissue regeneration [10], protect against tissue injury [11], or reduce skin aging [12]. With respect to the multifunctional bioactivities of polysaccharides, it is likely that the effect of St. John's Wort extracts is not only related to the main components. As the bioactivity of polysaccharides is closely related to their composition, water-soluble polysaccharides of St John's Wort were fractionized according to their acidity and analyzed concerning composition and linkage prior to the investigation of their activity on skin cells. Normal human dermal fibroblasts (NHDF), HaCaT, and normal human epidermal keratinocytes (NHEK) are useful tools to investigate different aspects of cell physiology in *in vitro* studies and allow the examination of underlying mechanisms. The investigations of the present study focused on the proliferation (BrdU-incorporation), the reductive enzyme activity (MTT and WST-1), involucrin and collagen expression and necrotic (extracellular lactate dehydrogenase activity) and apoptotic (Annexin V) effects of human skin cells. Mechanistical studies were carried out using real-time PCR and gene microarrays. As representative factors for RT-PCR, the fibroblast growth factor 7 (FGF-7), epidermal growth factor receptor (EGFR), insulin receptor, and the signal transducer and activator of transcription 6 (STAT6) were chosen as representative for proliferation related processes [13]. Regulation of differentiation on the gene level was assessed by phospholipase *α* (PLA2) [14] and involucrin [15]. An overlook over the affected signal pathways was obtained by gene microarray analysis of 1308 genes that refer to human skin.

## 2. Materials and Methods

### 2.1. Isolation and Characterization of Polysaccharides

250 g of powdered St. John's Wort herb from Caesar and Loretz GmbH, Bonn, Germany, was exhaustively extracted for 24 h in a Soxhlet extractor with acetone and methanol. The drug was identified by microscopic characterization, and a voucher specimen is deposited in the archive of the Institute for Pharmaceutical Biology and Phytochemistry (no. HP561/D). The residue was dried at room temperature and extracted 3 times each with 2 L Aqua Millipore under permanent stirring. The combined water extracts were concentrated using a rotary evaporator at 35°C and precipitated with ethanol 96% (V/V) to a final concentration of 80%. The entire precipitated polysaccharides (RPSs) were isolated by centrifugation at 3600 x g, dialyzed against Aqua Millipore using cellulose membranes (MWCO 3.5 kDa) for 5 days at 4°C, and lyophilized. Fractionation of RPS was performed according to the polysaccharides acidity by anion exchange chromatography on DEAE-Sephadex. Stepwise elution of polysaccharides was done with water and sodium phosphate buffers of increasing ionic strength (NaPB) according to Deters et al., 2005 [16]. For determination of the total carbohydrate, uronic acid and protein amounts polysaccharides were hydrolyzed with trifluoroacetic acid 2 mol/L at 121°C for 60 min and analyzed by TLC on silica gel F_254_ glass plates using acetonitrile-water 80 : 20 (V/V) as a mobile phase. After threefold development the monosaccharides were detected using 2-isopropyl-5-methylphenol/sulphuric acid spray reagent and heating at 120°C for 5 min. Reference standards for detection of neutral carbohydrates [17] and uronic acids [18] were prepared according to the monosaccharide and uronic acid composition determined via TLC. The protein contents were determined against reference concentrations of BSA (PAA, Laboratories, Coelbe) with Coomassie brilliant blue G250 [19]. Each of these tests was modified for use in 96-well-microtiter plates. A radial gel diffusion experiment using *β*-D-glucosyl-Yariv reagent [20] against gum arabic as reference (0.25 mg/mL, 0.5 mg/mL, 1 mg/mL, 2.5 mg/mL, 5 mg/mL, 10 mg/mL) was used to test the polysaccharide fractions for the occurrence of arabinogalactan proteins (AGP). Starch was assayed using Lugol's solution.

### 2.2. Linkage Analysis of Polysaccharides

Uronic acid was identified by ion-exchange HPLC with pulsed-amperometric detection (Bio-LC, Dionex, Idstein, Germany) with an AS50 autosampler, GS50 gradient pump, AS50 oven, and ED50 electrochemical detector on a CarboPacTM PA1 analytical column, 2 mm × 250 mm, CarboPacTM PA1, guard column 2 mm × 50 mm, and BorateTrapTM, 4 mm × 50 mm using a ternary gradient of water, 0.1 mM sodium hydroxide, and 0.5 mM sodium acetate. Neutral sugars were quantified by preparing trimethylsilyl derivatives: 10 mg of hydrolyzed polysaccharide was mixed with 1 mL TriSil-Z (Pierce, Bonn, Germany) and heated for two hours at 80°C. The separation and detection were done on a AGILENT Technologies 6890 system using an HP-PAS 1701 column (0.32 *μ*m × 25 m × 0.25 *μ*m) with helium as carrier gas and a temperature program from 160°C to 220°C with a heating-up rate of 10°C/min (injector: 275°C). Detection took place with a mass selective detector (70 eV ionization energy; 8 kV acceleration voltage), AGILENT Technologies, Santa Clara, USA). Structures of polysaccharides were analyzed according to the permethylation method of [21, 22], and the reduction of uronic acids with sodium borodeuteride was performed as described by [23]. Permethylated alditol acetates were injected at 220°C, gas chromatographed with a AGILENT Technologies 6890 N system on an HP-5MS column (0.25 mm × 30 m × 0.25 *μ*m) with helium as carrier gas and a temperature program from 170°C to 220°C with a temperature slope of 1°C/min. Mass fragments were detected with a mass selective detector (AGILENT Technologies, Santa Clara, USA) using 70 eV ionization energy and 8 kV acceleration voltage. The configuration of monosaccharides was determined by capillary electrophoresis versus D and L references using a P/ACE 5010 Instrument (Beckman Coulter, Krefeld, Germany) with an uncoated silica capillary (77 cm × 50 *μ*m) and DAD UV detector at 200 nm according to the method of Noe and Freissmuth, 1995 [24]. Polysaccharides were investigated in regard to esterification with IR-spectroscopy (mid-infrared) on a FT/IR-4100 type A (Jasco, Tokio, Japan) and a pectin C, which contained 70% ester bounds as reference.

### 2.3. Cell Culture

Confined cell lines were obtained after isolation of normal human dermal fibroblasts (NHDFs) and normal human epidermal keratinocytes (NHEKs) from human skin grafts (University Clinical Centre of Münster, Germany, Department of Dermatology, Department of Pediatrics) of various Caucasian subjects. The studies were approved by the local ethical committee of the University of Münster (acceptance no. 2006-117-f-S). Subculture of cells was done as described earlier [16, 25]. HaCaT keratinocytes, a friendly gift of Professor Fusenig, DKFZ, Heidelberg, were used for apoptosis-related experiments.

### 2.4. Cell Viability, Proliferation, and Differentiation

For investigation, polysaccharides were solved in Aqua Millipore to a concentration of 1 mg/mL, sterile filtered through a 0.2 *μ*m regenerated cellulose acetate membrane, and solved in the recommended serum-free media to a final concentration of 10 *μ*g/mL. As positive control, 10% FCS was added to test medium. All tests were realized in 96-well plates (Sarstedt, Nuembrecht, Germany) at starting cell densities of 5 × 10^3^ NHEK and 3 × 10^3^ NHDF in each well. Incubation with polysaccharides started 24 h after seeding when the cells had reached a confluence of 50% and ended 48 h later by adding BrdU and WST-1 reagents. BrdU incorporation assay and WST-1 and LDH assays were performed according to the manufacturer instructions (Roche Diagnostics, Penzberg, Germany). Activity of intracellular reducing enzymes was measured with MTT [26]. Apoptosis was measured after incubation of NHDF and NHEK with 10 *μ*g/mL polysaccharide by treating with Annexin V-PE and counterstaining with 7-aminoactinomycin (7-AAD) on a FACSCalibur flow cytometer (Becton Dickinson GmbH, Heidelberg, Germany). Cells were harvested by scraping in ice-cold Annexin V binding buffer, further proceeding following manufacture description. Differentiation of NHEK was elucidated by semiquantitative determination of involucrin amounts using the dot blot technique as described earlier [25].

### 2.5. Protein Expression of Cells

The collagen expression of fibroblasts was analyzed by a colorimetric method based upon Sirius Red. Therefore, NHDFs were seeded in 24-well cell culture plates (Greiner, Frickenhausen, Germany) with 7 × 10^4^ cells/well. At 70% confluence the cells were incubated with 10 *μ*g/mL polysaccharides solved in test medium, 100 mM L-ascorbic acid was used as positive control [27]. After 48 h, the culture supernatant (0.5 mL) was transferred to an Eppendorf cup and mixed with protease inhibitor mix (Roche Diagnostics, Penzberg, Germany). NHDFs were lysed by a fivefold freeze-thaw-cycle in 200 *μ*L 0.5 M acetic acid containing protease inhibitor mix and scraped from the plate. The resulting cell suspension was conveyed in a new Eppendorf cup. Solutions of cells and culture supernatants were stirred overnight at 4°C and afterwards centrifuged for 10 min at 14,000 x g to remove the cell debris. 50 *μ*L of the resulting solution was mixed with 450 *μ*L Sirius Red (69 *μ*g/mL, 0.5 M acetic acid), agitated for 30 min, and again centrifuged (10 min, 14 000 x g). Subsequently, the resulting pellet was solved in 50 *μ*L 0.1 M potassium hydroxide, and the intensity of color was determined at 540 nm with a reference wavelength of 690 nm.

### 2.6. Gene Expression Analysis of Keratinocytes and Fibroblasts by Real-Time PCR

Analysis of cellular signal transduction effected by St. John's Wort polysaccharides was done by gene expression analysis with real-time PCR after reverse transcription of total RNA. To exclude effects of FCS the fibroblasts were adapted to minimal MEM containing glucose  (4.5 g/L) and L-glutamine (1%) but no other supplements for 12 h. 10 *μ*g/mL polysaccharides and 10% FCS as positive control were solved in the minimal MEM prior to the incubation with NHDF for 6 h, 12 h, and 24 h. For untreated control cells fresh minimal MEM was used. After trypsinization of cells, the total RNA was isolated with the innuPREP RNA Mini Kit (Analytik Jena, Jena, Germany). After qualitative and quantitative analysis of obtained RNA with the biophotometer (Eppendorf, Hamburg, Germany) it was reverse-transcribed using the High-Capacity cDNA Reverse Transcription Kit. cDNA was diluted with RNAse-free water to 20 ng cDNA, and real-time-PCR was performed with TaqMan gene expression assays (Table 1) and the TaqMan Universal MasterMix, without AmpErase on a 7300 Real Time PCR System (all Applied Biosystems, Foster City, USA). The gene expression was calculated with the comparative Ct method.

### 2.7. Microarray Analysis of Gene Expression

For gene microarray analysis, NHDFs were seeded at a cell density of 5 × 10^5^ cells/75 cm^2^ cell culture flask (Sarstedt, Nuembrecht, Germany). At a confluence of 70% the medium was changed against minimal MEM, containing 4.5 g/L glucose as well as 1% L-glutamine and kept for 12 h to adapt cells to minimal culture conditions. For 24 h the cells were incubated with polysaccharides, solved in minimal MEM (10 *μ*g/mL), or only with fresh minimal MEM (control sample). After trypsinization the cells were washed with PBS and frozen in liquid nitrogen. Isolation and amplification of the RNA as well as the analysis of the gene expression via PIQOR Skin Microarray was performed by Miltenyi Biotech, Bergisch-Gladbach, Germany. For quality control the RNA integrity number (RIN) was determined with 8.2 (must be >6), indicating sufficient quality for gene expression experiments.

### 2.8. Statistical Analysis

Statistical evaluation was performed by Dunnett's post hoc test for comparison of three to four treatment groups after variance calculation by Levene. The results of three independent biological repeats were considered significant when the *P* value was less than 0.05. All data presented are the means of 24 random samples (errors bars: ±SE) or representative with *n* = 8.

## 3. Results

### 3.1. Isolation and Characterization of St. John's Wort Herb Polysaccharides

Carbohydrates were isolated from defatted St. John's Wort herb in a yield of 1.3% related to the starting material. The ochre brown product was assessed to be a polysaccharide with neutral sugar content of 78%, 16% of uronic acids, and a residual protein content of 6%. According to the ionic strength of the respective elution buffer and the acidity of polysaccharides, the AEC resulted in four polysaccharide fractions further referred to as Hp1, Hp2, Hp3, and Hp4. Main amounts of polysaccharides were eluted using 0.25 M NaPB (Hp3, 47%). High acidic (0.5 M NaPB; Hp4) and low acidic polysaccharides (0.1 M NaPB; Hp2) reached values of almost 23% each. 7% of polysaccharides were received by water elution (Hp1). The proportion of neutral sugars in the particular AEC fractions was determined as 93% (Hp1), 69% (Hp2), 63% (Hp3), and 47% (Hp4). By HPEAC-PAD uronic acids were identified and quantified as galacturonic acid and glucuronic acid. Protein analysis revealed protein amounts of 4% (Hp1), 6% (Hp2), 5% (Hp3), and 8% (Hp4). The reaction with Yarif-reagent showed that the protein amounts originate from arabinogalactan proteins (AGP) in fractions Hp2 (75%), Hp3 (3%), and Hp4 (3%) but not in fraction Hp1. Structural analysis was done after derivatization of hydrolyzed polysaccharides to silylated (TMS) derivates and permethylated alditol acetates (PMAA) followed by gas chromatographic separation and mass spectrometric detection. TMS derivatives were identified as arabinose, galactose, and glucose as well as rhamnose, xylose and mannose. Arabinose and galactose were present in a ratio of 1 : 1 in all fractions being the main monosaccharides in AGP containing fractions whereas Hp1 mainly contained glucose. Rhamnose was only present in amounts more than 10% in Hp2 and Hp4. Xylose and mannose were detected in minor amounts. In all fractions arabinose was found as terminal, (1→5), and 1,3,5 linked. The acidic fractions Hp2, Hp3, and Hp4 contained rhamnose linked in terminal, (1→2), and 1,2,3-linked forms. Xylose was linked in (1→2) position and terminal in Hp1. All fractions contained (1→4)-linked mannose and (1→4)-linked glucose but the test for starch was negative. Especially in Hp1 glucose was also linked in positions (1→6), terminal, (1→2), and 1,4,6. (1→3)-, (1→6)-, and 1,3,6-linked galactose was detected in all fractions as well as (1→4) linked glucuronic acid. Galacturonic acid was found as (1→3), (3→6), or 1,4,6 linked (Table 2). Capillary electrophoresis revealed an L-configuration of arabinose and D-configuration of xylose, galactose, and glucose. Infrared spectrometry revealed that the galacturonic containing fractions exhibited absorptions at wavenumbers 1233 cm^−1^ and 1733 cm^−1^ in consequence of a partial esterification of the galacturonic acid residues.

### 3.2. Influence of St. John's Wort Herb Polysaccharides on Cell Physiology of Human Keratinocytes

The proliferation of NHEK was marginally influenced by each polysaccharide fraction with less than 20%. Contrary to the polysaccharides of fractions Hp1, Hp2, and Hp4, the polysaccharides of Hp3 reduced the proliferation rates. MTT test revealed a slight but not significant increase of intracellular reducing enzymes activity after treatment of NHEK with all polysaccharide fractions. Necrotic effects of St. John's Wort herb polysaccharides were not observed on keratinocytes. Just weak apoptotic effects were observed with exception of HaCaT keratinocytes that were incubated with polysaccharides of fraction Hp3 (Table 3) whereas this effect was not significant.

After prolonged incubation time, NHEKs showed morphological changes in consequence of an incipient differentiation. For that after nine days the cellular proteins were extracted followed by a semi-quantitative determination of the differentiation-specific protein involucrin using dot blot technique.

**Figure 1:**
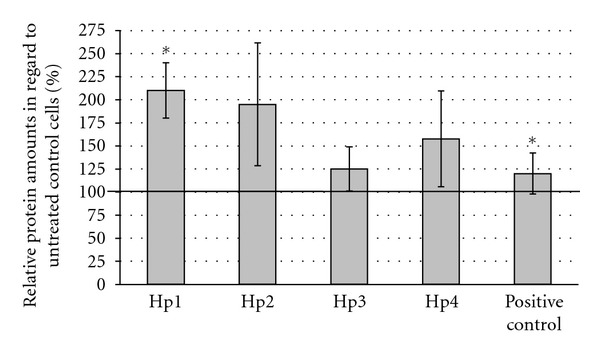
Expression of differentiation-specific protein involucrin of NHEK after treatment with 10 *μ*g/mL St. John's Wort polysaccharide fractions for nine days. Protein amounts were determined by semiquantitative dot blot technique. Obtained values with *n* = 8 were normalized to untreated NHEK (—: untreated NHEK = 100%). To induce the differentiation, the serum-starved MCDB 153 complete media were supplemented with 13 *μ*g/mL A23187 and 2 mM Ca^2+^ (positive control).

As shown in Figure 1 the involucrin expression was significantly enhanced by polysaccharides of Hp1 whereas the other polysaccharide fractions had no significant influence.

The large variations resulted from the different behavior of normal cells due to their diverse sources. First investigation of gene expression of differentiation-specific proteins and proliferation-related pathways indicated that after short incubation time of 6 h the involucrin gene expression is not altered independent of the used polysaccharide fraction for treatment. But in case of Hp1, which showed the most impact on the differentiation, the gene expression of proliferation-related genes like EGFR and InsR was inhibited. The decrease in PLA2 expression that is part of the Ca^2+^ signaling and for that involved in the differentiation process revealed that the differentiation of NHEK is induced in a different way (Table 4).

### 3.3. Reactivity of Normal Human Dermal Fibroblasts to Polysaccharides of St. John's Wort Herb

**Figure 2:**
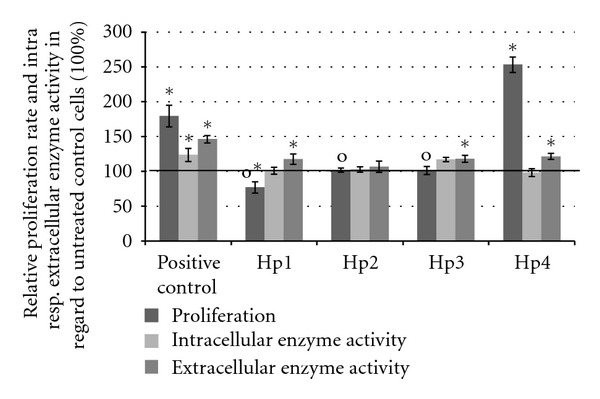
Results of relative proliferation (BrdU incorporation, representative with *n* = 8) and intracellular (MTT-reduction) and extracellular enzyme activity (WST-1 reduction) of NHDF treated with 10 *μ*g/mL St. John's Wort polysaccharides for 48 h. As positive control, 10% FCS was used. Values were normalized to untreated cells (—: untreated control = 100%). *n* = 24. Error bars = SE; *P < 0.05 compared to untreated cells and o: *P* < 0.05 to Hp4.

Polysaccharide fractions of St. John's Wort herb did not influence the activity of reducing enzymes in the cells as measured by MTT reduction assay. But as shown in Figure 2 the extracellular enzymes of Hp1-, Hp3-, and Hp4-treated NHDF reduced significantly more WST-1 than the untreated cells. The proliferation of NHDF was significantly stimulated by polysaccharide fraction Hp4 and reduced by high glucose amount containing fraction Hp1. Fractions Hp2, and Hp3 had no influence on the proliferation. Cytotoxic activity was not observed. Even the amount of necrotic cells was reduced compared to the untreated cells (0%): −2% in case of Hp2, Hp3 and Hp4 and −12% if NHDF. Apoptotic processes in NHDF were not affected by St. John's Wort polysaccharides. In Hp1-treated NHDF −12%  ±  10 of apoptotic cells were detected, NHDF incubated with Hp2, and Hp3 exhibited −8%  ±  6 respective −8%  ±  8 of apoptotic cells while Hp4 had no influence (1%  ±  24).

**Figure 3:**
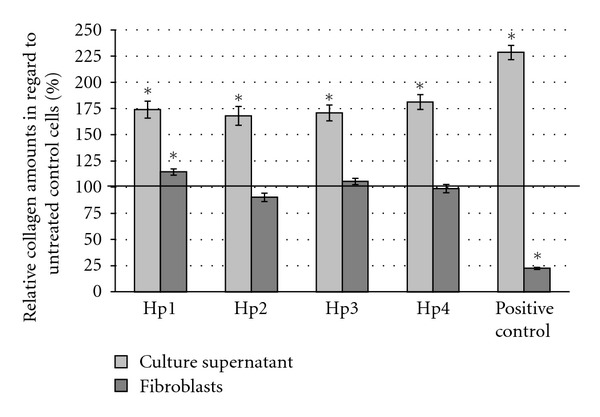
Relative amounts of acid soluble collagen of fibroblast culture supernatants and fibroblasts after incubation with 100 mM L-ascorbic acid as positive control and polysaccharide fractions (10 *μ*g/mL). Results were indirectly calculated by determination of hydroxyproline with Direct Red 80. Values shown are normalized to collagen amounts in supernatants and cells of untreated control (—: untreated control = 100%). *P < 0.05 compared to untreated cells; *n* = 4. Error bars = SE.

Collagen synthesis and release as parameter of fibroblast activity was measured indirectly by determination of hydroxyproline amounts with Direct Red 80 in cells and cell culture supernatant. Compared to untreated control fibroblasts the collagen release was significantly stimulated if NHDFs were incubated with each polysaccharide fraction but in less amounts compared to L-ascorbic-acid-treated NHDF (positive control). The amount of remaining intracellular collagen soluble within acetic acid was significantly increased if NHDFs were incubated with Hp1 but was not altered after incubation with polysaccharide fractions Hp3 and Hp4. Polysaccharides of Hp2 slightly reduced the intracellular acidic collagen amounts (Figure 3).

### 3.4. Influence on the Gene Expression of NHDF

NHDF were incubated for 6 h, 12 h, and 24 h with St. John's Wort herb polysaccharide fractions. The epidermal growth factor receptor (EGFR) was not affected by Hp3 and Hp4 independent of the incubation time while an increase in gene expression was observed after 12 h of incubation with Hp2. Hp1 repressed the EGFR gene expression after 6 h and 24 h of incubation. The gene expression of the fibroblast growth factor 7 (FGF7) depended on incubation time and used polysaccharide fraction. At 6 h of incubation the gene expression was reduced by Hp1 and not changed by the other fractions. If the incubation was prolonged for 6 h an upregulation occurred if the NHDFs were treated with Hp3 and Hp4 but after 24 h this effect inverted. Incubation of Hp2 resulted in a downregulation of FGF7 gene expression but only after 24 h. The gene expression of the signal transducer and activator of transcription 6 (STAT6) involved in the signaling of IL-4 was not changed by Hp2, Hp3, and Hp4 independent of incubation time. During the first 6 h of incubation Hp1 reduced the STAT6 gene expression but this effect was reverted if the incubation time was prolonged (Table 5).

Gene expression analysis by RT-PCR did not explain the activity of St. John's Wort polysaccharide fractions on NHDF. To get an idea which signal pathways were altered a microarray analysis was carried out. For the microarray analysis polysaccharides of AEC fraction Hp4 were chosen exemplarily because of their prominent effect on NHDF proliferation. DNA microarray indicated that the expression of 142 of 1308 genes in total changed after incubation for 24 h. But a significant regulation (>2 and <0.5 compared to untreated cells) was only found in case of 44 genes (Table 6). These genes were part of cellular processes like inflammation/stress response, transcription, metabolism, cell adhesion/extracellular matrix, and receptor signaling. Mostly an increase in gene expression occurred but the genes related to receptor signaling were predominantly downregulated. This coincides with the results of RT-PCR analysis concerning the gene expression of EGFR and KGF. Furthermore, the microarray revealed no influence on the cytokine signaling as it had been shown by RT-PCR with STAT6. In more detail prominent changes (>2) in gene expression were observed in case of genes that referred to cellular processes like inflammation (IKBE, FEN1, CXCL1), response to toxins (CMTM7, BGLAP), oxidative stress (SOD2, MUTYH), metabolism (NNMT), extracellular matrix (HSPG2), transcription (HOXD10, EXOSC10), cell motility (ACTG2) and of unknown function (LOC387763, C9ORF16). To a less extent but still significant was the up-regulation of genes referring to metabolism and transcription as well as cell adhesion and extracellular matrix as shown in Table 6. Additionally to genes related to receptor signalling (INHBC, CD16, GJB6, ZAP70) the gene expression of IL17A, C-FOS, AFM, CYP3A7, MMP7, and SPARCL1 was significantly decreased. No influence was observed concerning apoptosis, MAPK pathways, TNF signalling, mitochondria-associated metabolism, calcium signalling, cytoskeleton and translation-related processes.

## 4. Discussion

The presented results show that polysaccharide fractions denominated as Hp1, Hp2, Hp3, and Hp4 differ in acidity, monosaccharide composition, monosaccharide linkage, and their influence on human skin cells.

The polysaccharides belong to water-soluble hemicelluloses, nonswelling pectin, and arabinogalactan proteins (AGPs). In detail the most obvious difference was seen in fraction Hp1 that was composed of a high amount of glucose. Since the test with Lugol solution that only reacts with helical linked *α*-D-glucose, was negative, the glucose must be part of a *β*-D-glucan [28]. In regard to 1 : 1 ratio and linkage of galactose and arabinose a coexistence of an arabinogalactan is likely a result of the limited separation according to the polysaccharide acidity. For the same reason the acidic polysaccharide fractions Hp2, Hp3, and Hp4 would contain more than one polysaccharide. According to the positive reaction with the Yarif reagent, linkage of arabinose and galactose residues and their proportion pointed to the occurrence of arabinogalactan proteins (AGPs). Furthermore the ratio and linkage of arabinose, rhamnose, galactose, and galacturonic acid as well as the esterification of galacturonic acid indicates the presence of pectic structures. The increasing amounts of galacturonic acid and the different linkage types point to structural differences [29, 30]. To confirm these data the fractions must be purified and structurally characterized by NMR within future investigations.

Nevertheless the cell-based investigations showed that the polysaccharide fractions affect the skin cells corresponding to their composition and linkage of monosaccharide residues. Obvious was that polysaccharides of fraction Hp1 had the most efficient activity on NHEK differentiation and collagen synthesis of NHDF compared to the other polysaccharide fractions. According to previous investigations it is more likely that the *β*-D-glucan is responsible for this effect than the arabinogalactan. Arabinogalactans as well as AGPs have mostly shown to influence the proliferation of different cell types [7, 16, 30–32] while a similar *β*-D-glucan has proven to induce the differentiation of NHEK differentiation. We previously observed this effect in a study about Reed mace fruit polysaccharides [25]. Gene expression studies with keratinocytes and fibroblasts pointed to an influence of Hp1 on differentiation and proliferation processes by downregulation of genes for growth factors (FGF7), growth factor receptors (EGFRs), and insulin receptor (InsR), which are involved in promotion of proliferation and migration [13]. Especially a decreased EGFR signaling promotes epidermal differentiation [33]. Interestingly the differentiation was not influenced via PLA2 known to regulate the differentiation of keratinocytes [14]. Polysaccharide fractions Hp2, Hp3, and Hp4 were composed of AGP and pectic structures of different composition and linkage. AGPs as well as pectins are known to be biologically active in regard to their specific structure [30, 34]. The data of the present study does not allow to allocate the biologic activities neither to a specific AGP nor to a distinct pectic structure. However, it appeared that minor differences concerning linkage and amounts of monosaccharides are responsible for the different activity of these three polysaccharide fractions. Since differences in monosaccharide linkage will alter the structure of the whole polymer the polysaccharides of Hp4 differed from the polysaccharides of fractions Hp2 and Hp3. Treatment of fibroblasts with these three acidic fractions caused a stimulation of the proliferation rates only in case of fraction Hp4. Additionally, ANOVA analysis revealed that this effect was significant not only to untreated control cells but also to the effect of the other polysaccharide fractions. Gene expression analysis by RT-PCR revealed additional difference in their activity on cellular gene level. The effect of Hp4 polysaccharides on the fibroblast proliferation was not based on an influence on the gene expression of growth factors or their receptors as RT-PCR and microarray analysis revealed. At the investigated time point the predominant upregulation pertained to genes that refer to processes involved in oxidative stress (SOD2), response to toxins (CMTM7), and DNA repair (BGLAP). For that it is possible that the increased proliferation is due to a hyperproliferation as stress response. But the down regulation of connexin 30 (GJB6), a marker for hyperproliferation [35], and C-FOS, the lack of induction of inflammation-specific proteins like hypoxia inducible factor 1*α* (HIF1A), catalase (CAT), LPS binding protein (LPB), soluble epoxide hydrolase (EPHX2), chaperons and proinflammatory cytokines that are additionally part of the microarray contradict this hypothesis. Additionally the unaltered activity of reducing or antioxidative enzymes in the cells as determined with MTT test disagrees with a cellular response to oxidative stress.

The upregulation of the cytoplasmic malate dehydrogenase (MDH1) is interesting for its involvement in the carbohydrate metabolism. This and the fact that the other regulated genes are involved in transport mechanisms (CFTR, GLUT1, and AFM), drug metabolism (CYP3A7, PEG1-MEST, and NNMT) transcription processes (EXOSC10, HOXD10, SRRM2, ANKRD11, and MAZ) led to the hypothesis that the polysaccharides were internalized and catabolized by the cells and that the influence on the cells derives from inside and not outside the cell. According to the significant increase in extracellular enzyme activity the catabolism of polysaccharides may already start outside the cell followed by internalization. An internalization of an arabinogalactan with similar activity on cellular processes on gene level has been shown recently [32]. For that future studies must show if the polysaccharides were internalized or if they were degraded on the cell surface. Anyway the proliferation process is also influenced from outside the cell. The significantly upregulated genes of neuropilin (NRP1) and fibroblast growth factor 11 (FGF11) point to an influence of Hp4 polysaccharides on the FGF signaling [36]. Moreover, the proliferation process is influenced by other signal pathways as indicated by the regulation of perlecan (HSPG2), matrilysin (MMP7), and SPARC-like protein1 (SPARCL1). Another way to support the cell proliferation is to maintain cell adhesion and migration. So it is not surprising that the L1 adhesion molecule (L1CAM) and the cadherin-related tumor suppressor homolog (FAT) gene expression was affected by Hp4 polysaccharides. The regulation of gene expression may vary over the incubation time as mostly late signal pathway steps were affected. So shortening of incubation time for gene expression studies will show a different pattern of gene expression shifted towards earlier signaling processes and extracellular proteins [37]. The lacking influence on collagen expression coincides with unchanged intracellular collagen amounts indicating no increased or induced collagen synthesis.

Present results show that polysaccharides of different composition and linkage influence human skin cells in different ways. Gene expression studies support the physiologic data and reveal a foundation for the underlying mechanisms of polysaccharide activity.

## 5. Conclusion

Concluding, the water-soluble polysaccharides isolated and characterized from St. John's Wort herb are similar to already described water-soluble plant polysaccharides. Anyway the high *β*-D-glucose content and linkage of neutral AEC fraction Hp1 are characteristic for St. John's Wort herb and uncommon for already described polysaccharides of dicotyledons. Cell physiological investigations showed that the polysaccharides of Hp1 differ not only in their structure but also in their biological activity from the other extracted polysaccharides. Furthermore, the results obtained with acidic polysaccharides illustrate that slight structural differences lead to obvious differences in the biologic activity. However, future studies are necessary to determine the effective polysaccharide structure. Nevertheless, the presented data demonstrate that polysaccharides play a role in the effect of St. John's Wort extracts on skin cells.

## Figures and Tables

**Table 1:** TaqMan Assay IDs (Applied Biosystems, Foster City, USA) of specific proteins and representatives of signal pathways expressed in normal human fibroblasts (NHDFs) and normal human keratinocytes (NHEKs) investigated by real-time PCR after treatment with 10 *μ*g/mL St. John's Wort herb polysaccharide fractions.

Gene	Function	TaqMan Assay ID
Epidermal growth factor receptor (EGFR)	Proliferation	Hs01076068m1
Fibroblast growth factor 7 (FGF7)	Proliferation	Hs00384281_m1
Insulin receptor (InsR)	Survival/proliferation	Hs00169631m1
Involucrin (Inv)	Differentiation-specific protein	Hs00846307s1
Signal transducer and activator of transcription 6 (STAT6)	IL-4 signal transduction	Hs00598618_m1
Phospholipase *α* (PLA2)	Ca^2+^ signaling	Hs00179898_m1
Human 18s rRNA (18s)	Endogenous control	Hs99999901s1

**Table 2:** Results of methylation analysis and AGP determination of St. John's Wort polysaccharides. Data indicate the respective molar composition [%], calculated from results of HPAEC-PAD and silylation analysis. AGP amounts were detected by the agar diffusion test according to van Holst and Clarke, 1985 [20].

	Hp1	Hp2	Hp3	Hp4
1-Ara	6	23	6	1
1,2-Ara		1		1
1,3-Ara	3		2	
1,5-Ara	6	5	12	10
1,3,5-Ara	2	3	1	2
1,2,3-Ara			2	3
1,2,5-Ara	2		1	7
1,3,4,5-Ara			1	
Arapent			1	

1-Rha		1	3	4
1,2 Rha		1	2	2
3,4/1,4-Rha		9		2
1,2,3-Rha		1	2	6

1-Xyl	1			
1,2-Xyl	1	1		1
1,3,5-Xyl			1	

1-Man				1
1,4-Man	2	2	3	3
1,4,6-Man	1		1	

1-Glc	7			1
1,3-Glc	5			2
1,4-Glc	11	1	1	3
1,6-Glc	26		2	1
1,2,4-Glc	2			
1,4,6-Glc	3			1

1-Gal	1	1	1	
1,3-Gal	2	1	3	3
1,6-Gal	5	5	5	3
3,6-Gal	1		1	1
1,2,4-Gal			1	1
1,4,6-Gal	3	2		1
1,3,6-Gal	6	23	21	5
1,3,4,6-Gal	1		2	1

1,4-GlcAc	3	18	8	6

1,3-GalAc			17	
3,6-GalAc				15
1,3,6-GalAc		2		
1,2,4-GalAc				13

AGP amount [%]	0	75	3	3

**Table 3:** Influence of St. John's Wort polysaccharides on cell physiology of keratinocytes (NEHK and HaCaT). Results were normalized to the untreated cells calculated as 0%. Proliferation was determined by BrdU incorporation ELISA. MTT reduction assay was performed to investigate the intracellular enzyme activity. The cytotoxicity was determined by calculating the extracellular LDH activity compared to total LDH activity according to the manufacturer instructions. Apoptosis was measured by flow cytometry using Annexin V and 7-AAD.

Polysaccharide fraction	Proliferation rate (NHEK)	Intracellular enzyme activity (NHEK)	Amount of necrotic cells (NHEK)	Amount of apoptotic cells (HaCaT)
Hp1	16% ± 8	17% ± 7	0%	13% ± 11
Hp2	16% ± 9	23% ± 7	4%	15% ± 10
Hp3	−17% ± 8	21% ± 10	8%	21% ± 9
Hp4	10% ± 7	13% ± 6	5%	6% ± 3

**Table 4:** Gene expression of NHEK after 6 h of incubation with 10 *μ*g/mL St. John's Wort polysaccharides in serum-starved media. Results of RT-PCR with TaqMan Assays were normalized to endogenous control 18srRNA and to respective target gene expression of untreated control cells. Regulation was defined as significant with >2 (+) and <0.5 (−). No change in gene expression is shown as “o”.

	Involucrin	PLA2	EGFR	InsR
Hp1	o	−	−	−
Hp2	o	o	o	o
Hp3	o	o	o	o
Hp4	o	+	o	o

**Table 5:** Gene expression of NHDF after incubation with 10 *μ*g/mL St. John's Wort polysaccharide fractions in minimal MEM for 6 h, 12 h, and 24 h. Results of RT-PCR with TaqMan assays were normalized to endogenous control 18 srRNA and to the respected expression of target genes in untreated control cells. Regulation was defined as significant with >2 (+) and <0.5 (−). No change in gene expression is shown as “o”.

Polysaccharide fraction	EGFR	STAT6	FGF7	Incubation time
Hp1	−	−	−	6 h
Hp2	o	o	o	
Hp3	o	o	o	
Hp4	o	o	o	

Hp1	o	o	o	12 h
Hp2	+	o	o	
Hp3	o	o	+	
Hp4	o	o	+	

Hp1	−	o	−	24 h
Hp2	+	o	−	
Hp3	o	o	−	
Hp4	o	o	o	

**Table 6:** Summary of genes with significantly changed expression (>1.7, <0.5) from 1308 genes in total within gene expression analysis using topic-defined PIQOR skin microarray (Miltenyi Biotech, Cologne, Germany) from NHDF after treatment with polysaccharides of Hp4 (10 *μ*g/mL) compared to an untreated control (=1) for 24 h.

Gene	Description and function according to NCBI gene database	Relative regulation
		(SD %)
*Miscellaneous*		

LOC387763	LOC387763 protein (unknown)	2.70/9%
C9ORF16	C9ORF16 (unknown)	2.37/21%
ACTG2	Gamma-2-actin (cell motility)	2.01/29%
GJB6	Connexin 30 (hyperproliferation)	0.48/32%

*Cell adhesion and extracellular matrix*		

HSPG2	Perlecan	3.05/33%
L1CAM	L1 cell adhesion molecule	1.93/13%
FGF11	Fibroblast growth factor 11	1.93/13%
PTK7	Protein-tyrosine kinase 7	1.78/19%
FAT	Cadherin-related tumor suppressor	1.73/13%
SPARCL1	SPARC-like protein 1	0.48/22%
MMP7	Matrilysin	0.42/14%

*Metabolism*		

NNMT	Nicotinamide N-methyltransferase	2.83/17%
CA12_2	Carbonic anhydrase XII	1.98/11%
SLC20A1	Member of solute carrier family 20 (phosphate transporter)	1.89/10%
MDH1	Cytoplasmic malate dehydrogenase	1.84/21%
*CFTR*	cAMP-dependent chloride channel	1.80/4%
*GLUT1*	Solute carrier family 2 member 1 (facilitated glucose transporter)	1.78/29%
*PEG1-MEST*	Mesoderm-specific transcript homolog (mouse)	1.71/17%
*CYP3A7*	Cytochrome P450 3A7	0.47/23%
*AFM*	Afamin (*α*-albumin)	0.33/15%

*Receptor signaling*		

NRP1	Neuropilin-1 (CD304 Antigen)	1.79/26%
INHBC	Inhibin *β*C	0.46/19%
CD16	IGG FC receptor III-2	0.43/21%
ZAP70	Zeta-chain (TCR) associated protein kinase 70 kDa	0.41/24%

*Transcription*		

EXOSC10	Exosome component 10	2.96/37%
HOXD10	Homeobox D10	2.04/20%
SRRM2	Serine/arginine-rich splicing factor-related nuclear matrix protein of 300 kDa	1.92/33%
MAZ	MYC-associated zinc finger protein (purine-binding transcription factor)	1.91/17%
ANKRD11	Ankyrin repeat domain 11	1.81/23%
MRPL28	Mitochondrial ribosomal protein L28	1.74/15%
C-FOS	G0/G1 switch regulatory protein 7	0.21/1%

*Inflammation, stress, and DNA repair*		

SOD2	Mitochondrial superoxide dismutase 2	4.78/22%
CMTM7	CKLF-like MARVEL transmembrane domain-containing protein 7	3.86/38%
CXCL1	Chemokine (C-X-C motif) ligand 1	3.33/18%
IKBE	Nuclear factor of kappa light polypeptide gene enhancer in B-cells inhibitor, epsilon	3.18/38%
FEN1	Flap structure-specific endonuclease 1	2.34/11%
BGLAP	Bone gamma-carboxyglutamate (gla) protein	2.07/10%
MUTYH	A/G-specific adenine DNA glycosylase	2.05/20%
BTF2P44	Basic transcription factor 2 44 kDa subunit	1.99/18%
IKBA	Nuclear factor of kappa light polypeptide gene enhancer in B-cells inhibitor, alpha	1.89/5%
SERPIN 1	Serine proteinase inhibitor	1.83/6%
XBP1	X-box binding protein 1	1.73/12%
HSP90B1	Endoplasmin (heat shock protein 90 b1)	1.73/21%
POLH	Polymerase eta (DNA directed)	1.70/20%
IL17A	Interleukin 17	0.45/20%

SD: standard deviations from *n* = 4 replicates of three biologic repeats.
